# Ecological structuring of biosynthetic potential across terrestrial soil-associated *Streptomyces* genomes

**DOI:** 10.3389/fmicb.2026.1884766

**Published:** 2026-10-21

**Authors:** Hajar Mhammedi, Sajida Mhammedi, Said Bouda, Youssef El Hilali Alaoui, Adil Essarioui

**Affiliations:** 1 Laboratory of Agro-Industrial and Medical Biotechnology, Faculty of Sciences and Techniques, Sultan Moulay Slimane University, Beni-Mellal, Morocco; 2 Oasis System Research Unit, National Institute of Agricultural Research, Regional Center of Agricultural Research of Errachidia, Rabat, Morocco; 3 Laboratory of Engineering and Research in Artificial Intelligence (LIRIA), National School of Artificial Intelligence and Digitalization (ENIAD), Mohammed First University, Berkane, Morocco

**Keywords:** biosynthetic gene clusters, ecological clustering, environmental metadata, genome mining, secondary metabolism, *Streptomyces*

## Abstract

*Streptomyces* populations represent one of the richest reservoirs of microbial specialized metabolites, although the ecological organization of their biosynthetic diversity remains insufficiently resolved at large genomic scales. In the present study, 14,597 publicly available genome assemblies were initially screened. Environmental metadata associated with each genome were subsequently retrieved from the corresponding NCBI BioSample records and systematically curated to retain genomes originating from terrestrial soil-associated environments while excluding marine, freshwater, clinical, laboratory, industrial, and other non-terrestrial sources. Standardized climatic and edaphic variables were examined using principal component analysis (PCA), K-means clustering, and Kruskal–Wallis tests to evaluate ecological differentiation across terrestrial soil-associated *Streptomyces* populations, with false discovery rate (FDR) adjustment applied to inter-cluster comparisons. PCA revealed pronounced environmental structuring, with Principal Component 1 (PC1) explaining 44.3% of the total variance and Principal Component 2 (PC2) contributing 31.1%, together accounting for 75.4%. K-means clustering resolved three environmentally coherent groups with distinct soil and climatic profiles. Significant differentiation was detected for soil pH, rainfall, temperature, soil organic carbon, and clay content, with adjusted *p*-values below 0.001. Genome mining using GECCO (GEne Cluster prediction with COnditional Random Fields) revealed extensive biosynthetic diversity dominated by polyketide- and non-ribosomal peptide-associated gene clusters. Total biosynthetic richness also differed significantly among ecological groups (Kruskal–Wallis statistic = 7.28, *p* < 0.05). This study provides a large-scale ecological-genomic framework linking environmental differentiation with biosynthetic organization across terrestrial soil-associated *Streptomyces* populations, advancing understanding of soil microbial and metabolic diversity.

## 1 Introduction

Members of the genus *Streptomyces* are among the most extensively studied soil-associated bacteria because of their remarkable capacity to produce structurally diverse secondary metabolites, including antibiotics, antifungals, immunosuppressive compounds, and numerous bioactive natural products (Bai et al., 2026; Connolly et al., 2026). Their large genomes typically encode extensive biosynthetic repertoires, often exceeding those observed in many other bacterial lineages (Dinglasan et al., 2025). This metabolic richness has positioned *Streptomyces* as a major focus of natural product research and microbial biotechnology for several dècades (Asokan et al., 2026; Buzón-Durán et al., 2025; Ly et al., 2026).

Several previous studies have explored the biosynthetic potential of *Streptomyces* using comparative genomics and genome mining approaches (Alam et al., 2022; Opoku Gyamfi et al., 2026). Early large-scale analyses mainly focused on the identification and classification of biosynthetic gene clusters (BGCs), demonstrating that *Streptomyces* genomes harbor extensive cryptic metabolic diversity with substantial natural product discovery potential. For example, comparative genomic investigations revealed that many *Streptomyces* strains contain large numbers of silent or poorly characterized BGCs that remain experimentally unexplored (Chung et al., 2021). Genome mining frameworks such as antiSMASH and GECCO have subsequently enabled scalable prediction of polyketide, non-ribosomal peptide, terpene, and RiPP-associated biosynthetic architectures directly from genome assemblies (Blin et al., 2025; Carroll et al., 2021; Guerrero Garzón et al., 2025; Jeong et al., 2025; Liu et al., 2025; Passari et al., 2025; Vignolle et al., 2025).

Coupled with recent advances in synthetic biology and genetic engineering, including CRISPR-based genome editing, biosynthetic gene cluster activation, pathway refactoring, and optimized heterologous expression systems, these approaches have accelerated the discovery, characterization, and industrial-scale production of secondary metabolites from microbial resources (Krysenko, 2025).

More recent studies expanded these approaches using pangenome-scale analyses to investigate evolutionary patterns and biosynthetic diversity across large collections of *Streptomyces* genomes. These analyses demonstrated that biosynthetic repertoires are highly dynamic and unevenly distributed among lineages, suggesting substantial ecological and evolutionary diversification (Mohite et al., 2025; Sudharsan et al., 2026). Other investigations combined genome mining with metabolomics or molecular networking to characterize novel compounds from environmental isolates, including strains recovered from marine sediments, mangroves, Antarctic soils, and unexplored terrestrial habitats (Du et al., 2024; Jiang et al., 2025; Shen et al., 2024).

Despite these advances, most previous studies primarily emphasized biosynthetic discovery, strain characterization, or evolutionary genomics. Comparatively limited attention has been directed toward understanding how biosynthetic diversity is distributed across ecological space. Environmental metadata associated with public microbial genomes also remain highly fragmented and inconsistently annotated, which restricts ecological interpretation at large scales. Consequently, relationships between environmental differentiation, ecological clustering, and genome-resolved biosynthetic organization across environmentally distributed *Streptomyces* populations remain insufficiently explored (Richard and Poulicard, 2025). In the present study, we explored ecological structuring associated with biosynthetic diversity across terrestrial soil-associated *Streptomyces* populations using a large-scale ecological-genomic approach. Publicly available genomes were curated alongside environmental metadata describing soil and climatic conditions, including soil pH, rainfall, temperature, soil organic carbon, and clay content. Multivariate ecological analyses, including principal component analysis (PCA), K-means clustering, and Kruskal–Wallis statistical testing, were subsequently applied to characterize environmental differentiation across sampling sites and identify environmentally coherent ecological groups. To further assess ecological-genomic relationships, genome mining was performed using the GECCO framework to predict biosynthetic gene clusters and evaluate variation in biosynthetic richness and metabolic architecture among ecological groups. Unlike previous studies primarily focused on comparative genomics or natural product discovery, this study integrates large-scale environmental metadata, multivariate ecological analyses, and genome-wide biosynthetic predictions within a unified ecological-genomic framework to investigate how ecological differentiation is associated with biosynthetic organization across environmentally associated *Streptomyces* populations.

## 2 Materials and methods

### 2.1 Genome dataset collection

Publicly available *Streptomyces* genome assemblies were retrieved from the National Center for Biotechnology Information (NCBI) RefSeq database using combinations of the terms “*Streptomyces*,” “genome,” and “complete.” Both complete genomes and high-quality draft assemblies were considered for downstream analyses. A total of 14,597 assemblies were initially screened for assembly quality and metadata availability.

Records associated with clinical isolates, industrial strains, laboratory-adapted cultures, or poorly described sources were excluded to retain environmentally relevant genomes. Each assembly was subsequently treated as an independent genomic unit throughout the study.

### 2.2 Metadata curation

Environmental metadata associated with each genome assembly were retrieved from the corresponding NCBI BioSample records. Multiple metadata descriptors were systematically examined, including geographic origin, isolation source, environmental biome, environmental feature, environmental medium, host association, and other available sample description fields.

To construct a dataset representative of terrestrial soil-associated *Streptomyces*, BioSample records were curated to retain only genomes originating from terrestrial environments. Records describing soil-associated habitats, including agricultural soils, forest soils, rhizosphere, plant-associated soils, compost, desert soils, mangrove soils, and other terrestrial substrates, were retained for downstream analyses. Genome assemblies originating from marine, freshwater, aquatic, clinical, human-associated, animal-associated, laboratory, industrial, or other non-terrestrial environments were excluded. Records containing incomplete environmental annotations, ambiguous habitat descriptions, or insufficient metadata to confidently assign a terrestrial origin were also removed during the curation process. Following environmental filtering, 895 environmentally annotated terrestrial strains were retained (Table 1). Additional quality filtering based on the completeness of environmental metadata reduced the ecological-genomic dataset to 364 high-quality genomes, of which 208 contained complete measurements for all selected environmental variables and were therefore retained for the final ecological analyses.

**Table 1:** Dataset curation and environmental filtering workflow.

Processing step	Number of records
Total *Streptomyces* assemblies screened	14,597
Environmentally annotated strains	895
High-quality environmentally enriched strains	364
Complete environmental strain records	208
Independent ecological sites	89

To avoid pseudoreplication, strain-level records sharing the same geographic coordinates were collapsed into a single ecological site, and median values were calculated for the environmental variables of strains originating from the same location. This site-level aggregation resulted in 89 independent ecological sites used for the environmental analyses.

### 2.3 Environmental variables

Environmental analyses were conducted using five ecological variables selected according to metadata completeness and ecological relevance: soil pH measured in water (*soil_phh2o*), soil organic carbon (*soil_soc*), soil clay content (*soil_clay*), mean annual temperature, and annual rainfall. These variables represented complementary climatic and edaphic gradients across sampled habitats. Environmental descriptors containing extensive missing values were excluded prior to downstream multivariate analyses to improve dataset consistency and statistical robustness.

### 2.4 Environmental metadata processing

Before multivariate analyses, environmental variables were evaluated for distributional heterogeneity and scale imbalance. Raw ecological measurements displayed substantial variation in numerical range and dispersion, particularly for rainfall and soil organic carbon. Preprocessing procedures were therefore applied to reduce the influence of extreme values and improve comparability among environmental descriptors (Mhammedi et al., 2023).

#### 2.4.1 Log transformation

Variables exhibiting pronounced positive skewness were log-transformed before downstream statistical analyses. Soil organic carbon (soil_soc) and annual rainfall showed strongly right-skewed distributions characterized by a limited number of highly elevated observations relative to the majority of sampling sites. Logarithmic transformation was applied to reduce variance asymmetry and moderate the disproportionate contribution of extreme environmental values during ordination analyses (Williams et al., 2023).

#### 2.4.2 Variable standardization

Continuous environmental variables were standardized using z-score normalization prior to principal component analysis and ecological clustering (Abdelshafy Mohamad et al., 2024). Standardization minimized scale-dependent biases among environmental variables measured using different numerical ranges.

### 2.5 Correlation analysis

Pairwise correlations among environmental variables were calculated using Pearson correlation coefficients prior to multivariate ordination analyses (Lee et al., 2025). Correlation analyses were performed on the processed environmental matrix to evaluate relationships among climatic and edaphic descriptors and to characterize covariance patterns across the dataset.

### 2.6 Principal component analysis

Principal component analysis (PCA) was applied to the standardized environmental matrix to characterize multivariate ecological variation across sampling sites (Greenacre et al., 2022). This ordination approach reduced dataset dimensionality by transforming correlated environmental descriptors into orthogonal principal components representing major sources of ecological variance.


$$PC_{i}=a_{i1}x_{1}+a_{i2}x_{2}+\ldots +a_{\mathrm{ip}}x_{p}$$

where ( $PC_{i}$ ) corresponds to the ( $i^{\mathrm{th}}$ ) principal component, ( $a_{\mathrm{ij}}$ ) denotes variable loadings, and ( $x_{j}$ ) represents standardized environmental variables.

The first two principal components were retained for visualization because they explained the majority of environmental variance across the dataset.

Environmental loadings were projected onto the ordination space to generate PCA biplots and visualize the relative contribution of environmental variables to principal component structure. Variable vectors were scaled according to loading magnitude and direction within multivariate environmental space.

### 2.7 Ecological clustering using K-means clustering

Ecological clustering was performed using K-means clustering on the standardized environmental dataset. The algorithm partitions observations into (k) ecological groups by minimizing within-cluster variance (Ikotun et al., 2023).


$$J=\sum_{i=1}^{k}\sum_{x_{j}\in C_{j}}{\left\|x_{j}-\mu_{i}\right\|}^{2}$$

where ( $C_{j}$ ) represents cluster (i), ( $x_{j}$ ) corresponds to individual observations, and ( $\mu_{i}$ ) denotes the cluster centroid.

The optimal number of ecological clusters was determined using silhouette analysis across multiple candidate values of (*k*). Site-level clustering was ultimately performed using (*k* = 3), which provided the strongest balance between cluster separation and ecological interpretability.

Environmental differentiation among clusters was assessed using Kruskal–Wallis non-parametric tests because several variables deviated from normality assumptions. Multiple-testing correction was performed using false discovery rate (FDR) adjustment.

### 2.8 Biosynthetic gene cluster prediction

Biosynthetic gene clusters (BGCs) were predicted using GECCO (Carroll et al., 2021), a machine-learning genome mining framework based on conditional random fields (CRFs) for *de novo* identification of biosynthetic gene clusters. GECCO was selected because, in addition to predicting BGC boundaries and biosynthetic classes, it provides probabilistic confidence scores for every predicted cluster, enabling quantitative assessment of prediction reliability across large genome collections. Genome assemblies retrieved from the NCBI RefSeq database were processed individually to preserve genome-level biosynthetic resolution during downstream ecological-genomic analyses. Predicted clusters were classified into major biosynthetic categories, including non-ribosomal peptides (NRPs), polyketides, RiPPs, terpenes, saccharides, alkaloids, and hybrid biosynthetic architectures. A total of 363 genomes successfully completed the GECCO analysis and were retained for subsequent ecological-genomic integration analyses (Eddy, 1996; Dağgeçen and Alptekin, 2026).

### 2.9 Ecological-genomic integration

Environmental metadata, ecological cluster assignments, and genome-resolved biosynthetic predictions were integrated into a unified ecological-genomic matrix using assembly accession identifiers as shared genome-level keys.

The resulting dataset incorporated environmental descriptors, multivariate ecological structure, biosynthetic richness estimates, and class-specific BGC distributions for each genome retained after quality filtering procedures.

### 2.10 Biosynthetic richness analysis

Biosynthetic richness was quantified as the total number of predicted biosynthetic gene clusters identified per genome. Richness estimates were subsequently compared among ecological clusters generated from multivariate environmental analyses.

Differences in total BGC richness across ecological groups were evaluated using Kruskal–Wallis non-parametric statistical testing because biosynthetic count distributions did not satisfy assumptions of normality.

### 2.11 Biosynthetic class composition

Class-specific biosynthetic composition was evaluated by quantifying the abundance of major BGC categories within each ecological cluster. Comparative analyses focused on dominant biosynthetic classes, including polyketides, NRPS-associated clusters, RiPPs, and terpene-associated regions. Mean class abundance profiles were calculated at the ecological cluster level and subsequently visualized using ecological-genomic comparative plots generated from the integrated biosynthetic dataset.

## 3 Results and discussion

This section presents the environmental structuring and biosynthetic profiling of terrestrial soil-associated *Streptomyces* genomes. Multivariate ecological analyses were applied to characterize major environmental gradients and identify ecologically differentiated groups across sampling sites. Biosynthetic richness and class-specific BGC distributions were subsequently integrated with ecological metadata to evaluate relationships between environmental differentiation and specialized metabolic diversity. All computational analyses, statistical procedures, genome mining workflows, and figure generation steps were conducted using reproducible Python-based pipelines on a workstation equipped with 16 GB RAM and a 3.20 GHz processor.

### 3.1 Environmental structure of independent ecological sites

The curated ecological dataset encompassed a broad range of climatic and edaphic conditions across environmentally associated *Streptomyces* habitats (Table 2). Soil pH values extended from moderately acidic to alkaline environments, whereas annual rainfall ranged from arid systems to highly humid ecological regions. Soil organic carbon and clay content also displayed substantial variability.

**Table 2:** Environmental characteristics of independent ecological sites.

Variable	Mean ± SD	Minimum	Maximum
Soil pH	6.31 ± 0.87	4.5	8.3
Soil organic carbon	55.47 ± 39.99	8.0	273.5
Soil clay	232.66 ± 81.63	75.0	500.0
Temperature (°C)	14.23 ± 7.55	−5.2	27.74
Rainfall (mm)	1002.25 ± 598.70	20.1	2967.5

Rainfall exhibited the largest overall dispersion within the dataset, varying from 20.1 mm to 2967.5 mm annually. Similarly, soil organic carbon values ranged from nutrient-poor environments to highly enriched soils. Temperature gradients further reflected the ecological breadth represented within the dataset, spanning subzero conditions as well as warm temperate environments.

Pairwise correlation analysis revealed structured relationships among several environmental descriptors (Figure 1). Soil pH showed a pronounced negative correlation with rainfall (*r* = −0.67) and a moderate inverse relationship with soil organic carbon (*r* = −0.53). In contrast, rainfall was positively associated with soil organic carbon content (*r* = 0.37). Temperature displayed moderate negative relationships with soil organic carbon, whereas clay content exhibited partial covariance with temperature-related gradients. Although several variables displayed coordinated environmental structure, none approached complete collinearity (Schniete and Fernández-Martínez, 2024).

**Figure 1:**
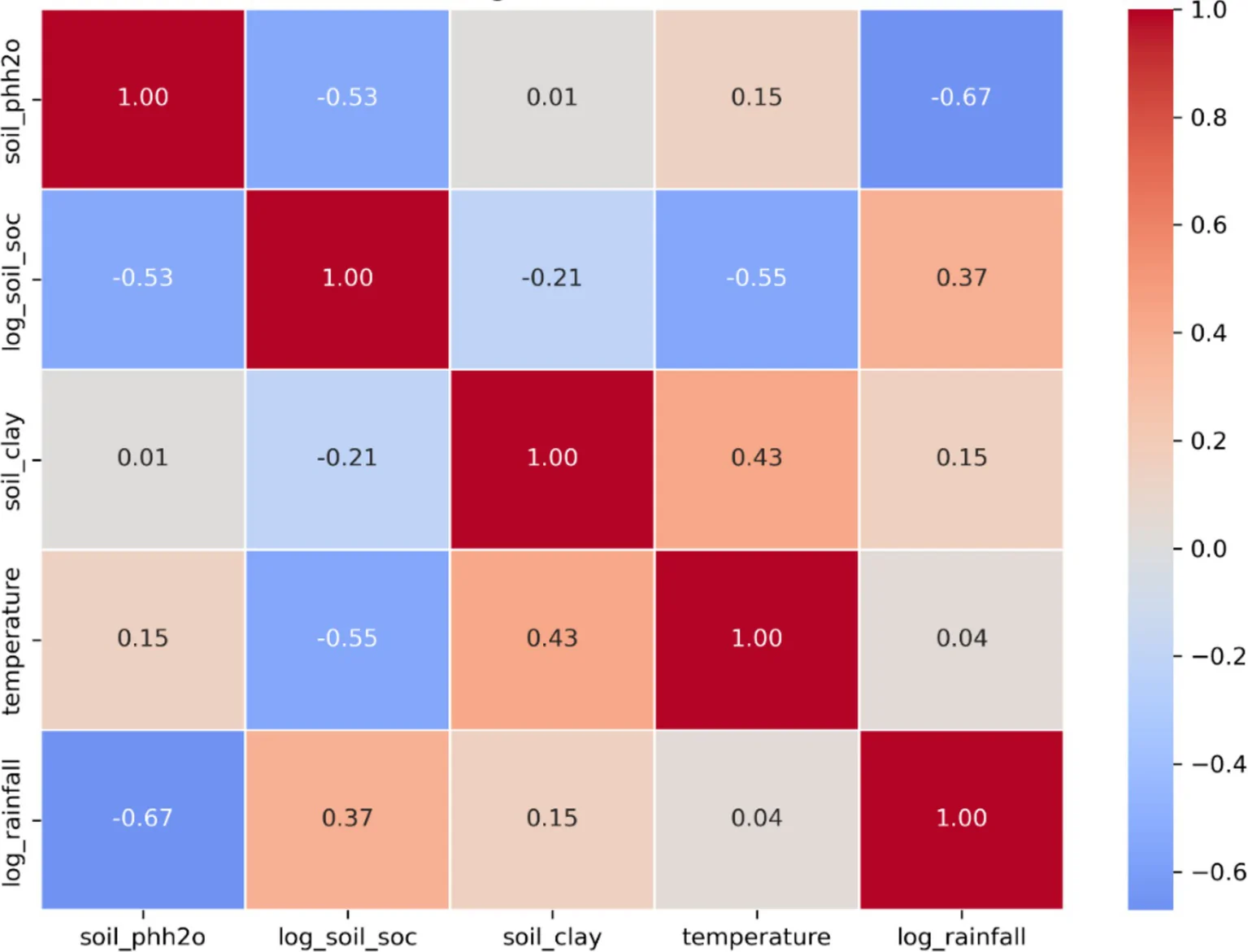
Pairwise correlation matrix. Soil pH measured in water (soil_phh2o), soil organic carbon (log_soil_soc), soil clay content (soil_clay), temperature, and annual rainfall (log_rainfall).

### 3.2 Principal component analysis of environmental gradients

Principal component analysis revealed pronounced environmental structuring across independent ecological sites associated with *Streptomyces* genomes. The first two principal components explained 75.4% of the total environmental variance, with PC1 accounting for 44.3% and PC2 contributing 31.1%.

The PCA ordination demonstrated that ecological sites were distributed along broad environmental gradients rather than forming a single homogeneous cluster (Figure 2). Several sites occupied intermediate environmental positions, whereas others extended toward more distinct climatic and edaphic conditions. Projection of environmental loadings onto the ordination space further clarified the variables contributing to ecological separation (Figure 3). Rainfall and soil organic carbon displayed similar directional orientations along positive PC1 values, while soil pH was positioned along the opposite axis, separating comparatively alkaline habitats from wetter environments. Temperature and clay content contributed primarily to positive PC2 space and represented an additional dimension of environmental differentiation within the dataset.

**Figure 2:**
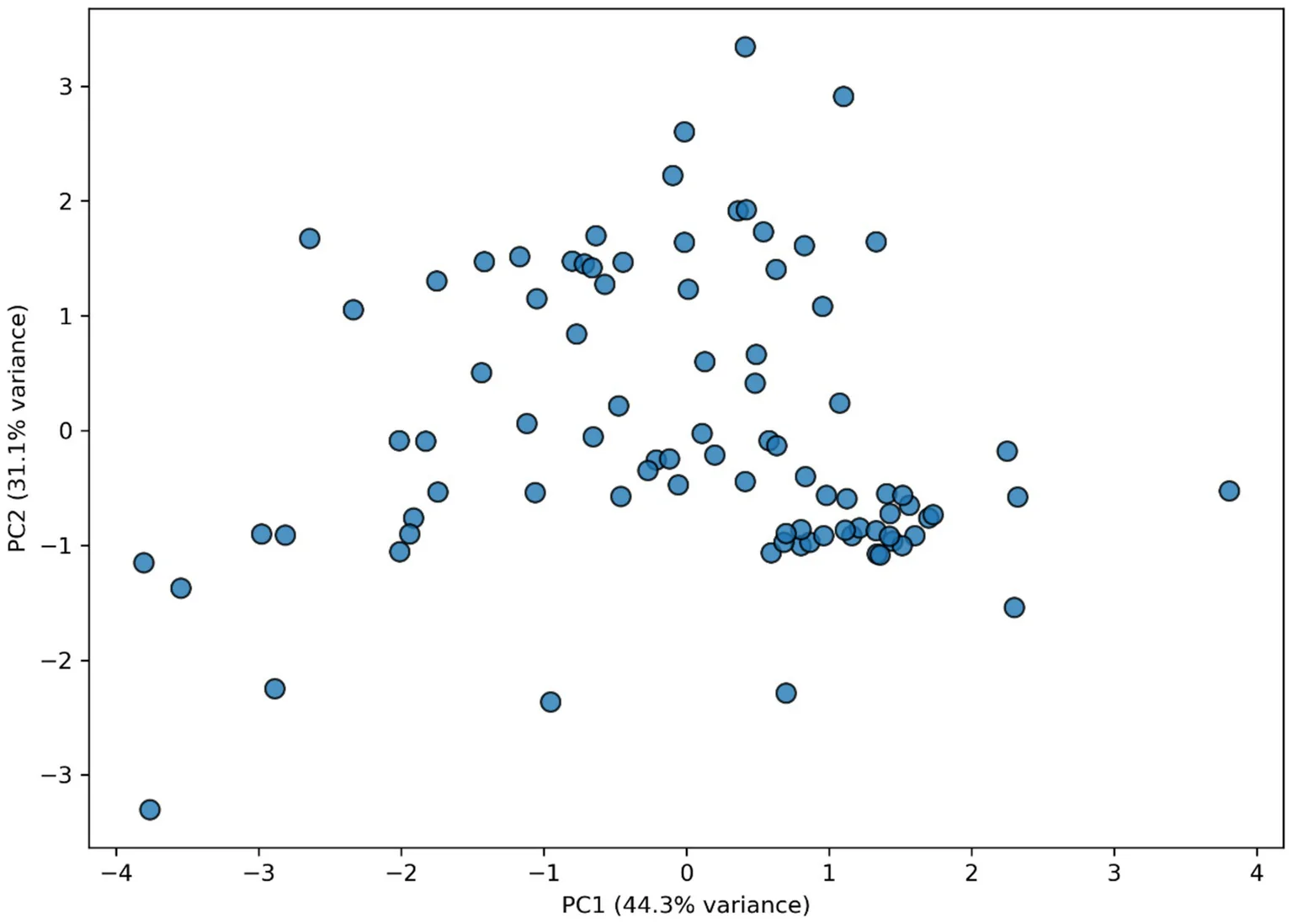
Principal component analysis (PCA) ordination of independent *Streptomyces* ecological sites based on standardized environmental variables. Each point represents an independent ecological site positioned according to multivariate environmental similarity.

**Figure 3:**
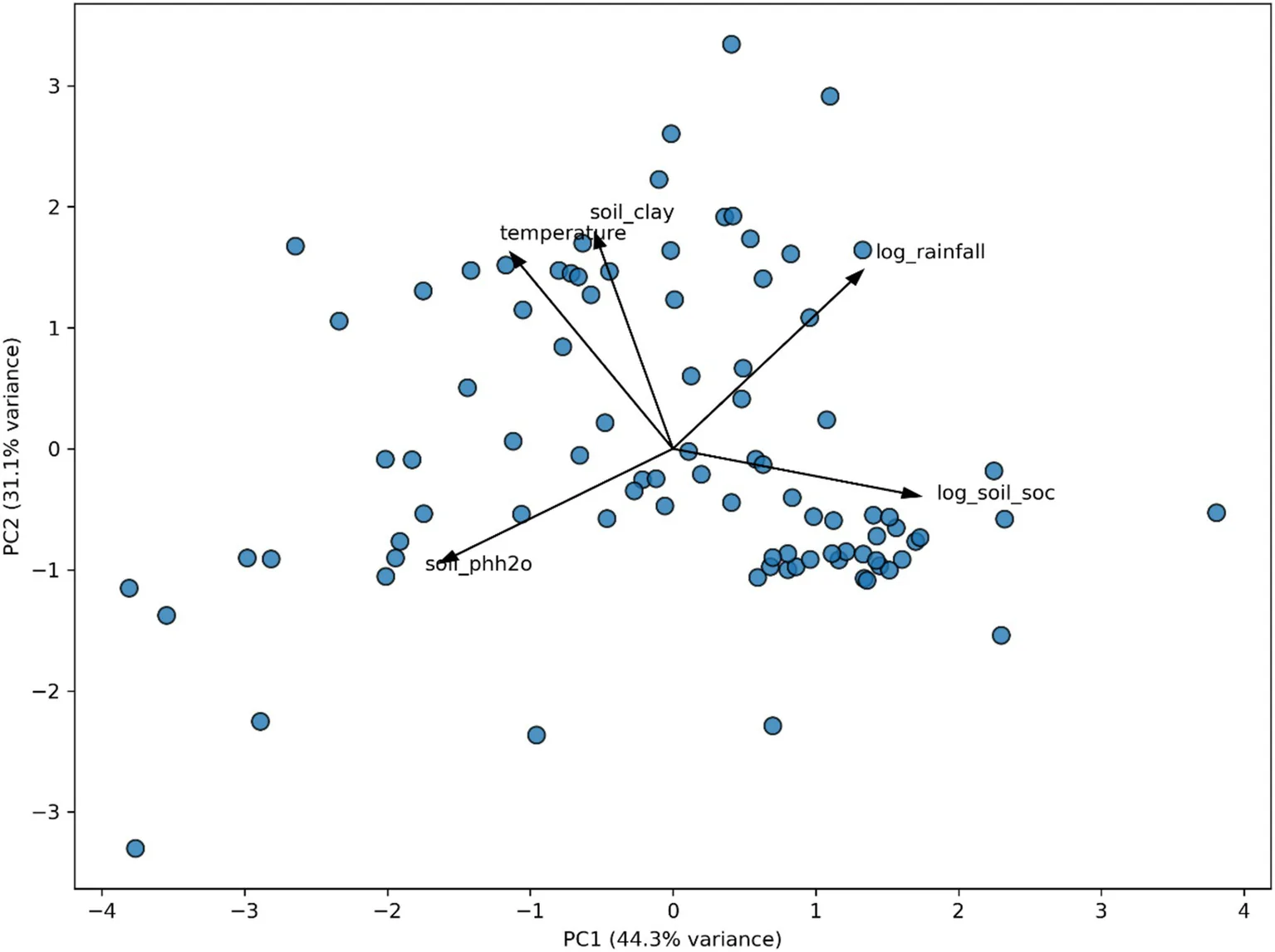
PCA biplot of environmental gradients across independent ecological sites.

### 3.3 Ecological cluster identification and environmental differentiation

K-means clustering resolved the environmental dataset into three major ecological groups occupying distinct regions of multivariate ordination space (Figure 4). Cluster separation was primarily structured along PC1, although additional differentiation remained evident across PC2. Distinct environmental signatures characterized each ecological cluster (Figure 5). Cluster 0 was mainly associated with elevated clay content, higher rainfall, and comparatively warm conditions. In contrast, Cluster 1 occupied environments enriched in soil organic carbon and displayed lower temperature and reduced clay-associated profiles. Cluster 2 differed substantially from the remaining groups and was primarily associated with alkaline soils and lower rainfall conditions.

**Figure 4:**
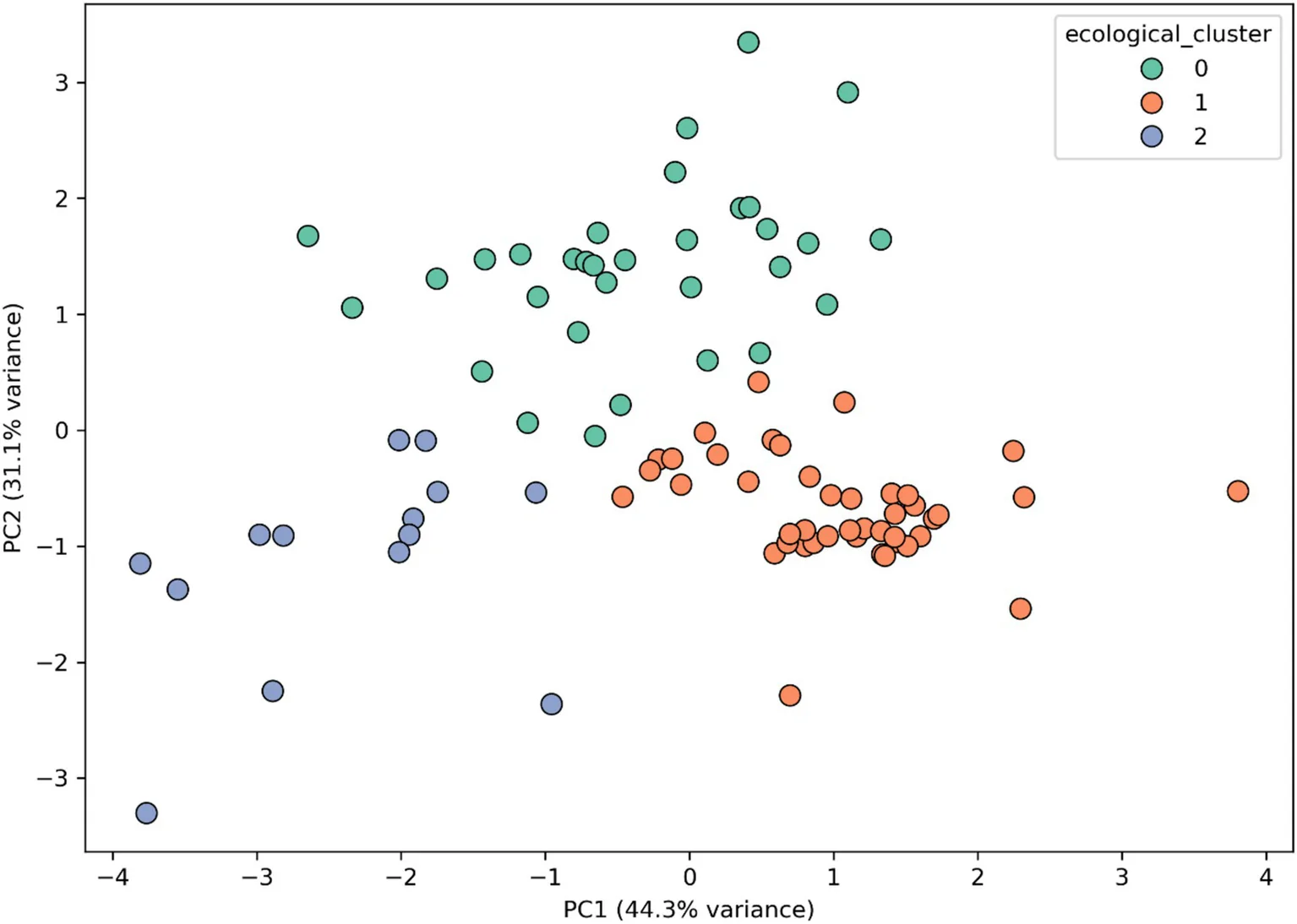
PCA-based visualization of ecological clusters identified by K-means clustering.

**Figure 5:**
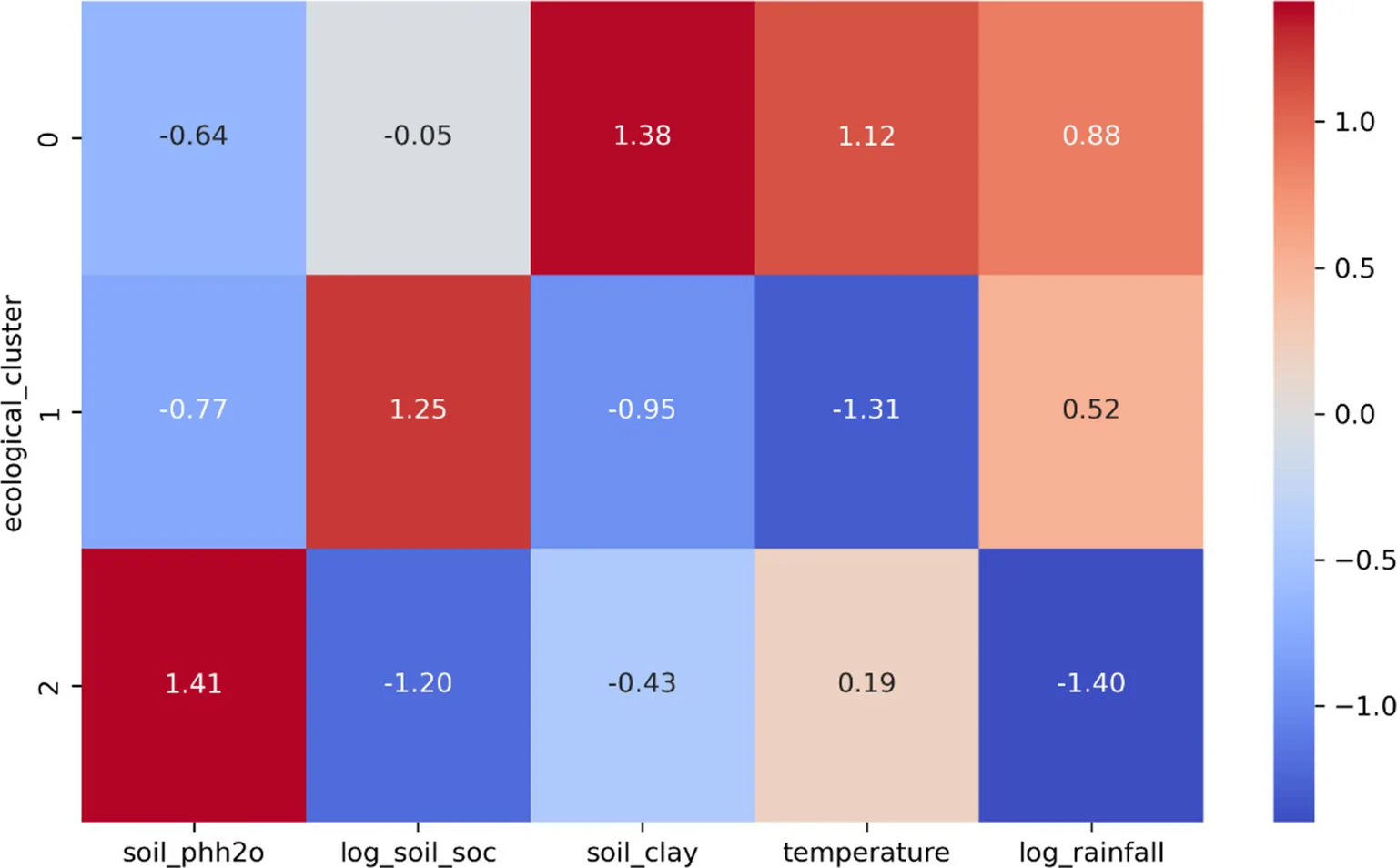
Standardized environmental profiles associated with ecological clusters. The identified ecological groups therefore represent environmentally coherent site assemblages structured by combined soil and climatic variation.

The environmental separation observed in multivariate space was further supported by the distribution of individual ecological variables (Figure 6). Soil pH values remained consistently elevated within Cluster 2, whereas Cluster 1 displayed higher soil organic carbon concentrations relative to the remaining ecological groups. Clay-associated environments were more strongly represented within Cluster 0, while rainfall distributions reflected differentiation between humid and comparatively arid ecological contexts. Temperature gradients contributed additional separation among clusters and reinforced the presence of distinct environmental profiles across the dataset.

**Figure 6:**
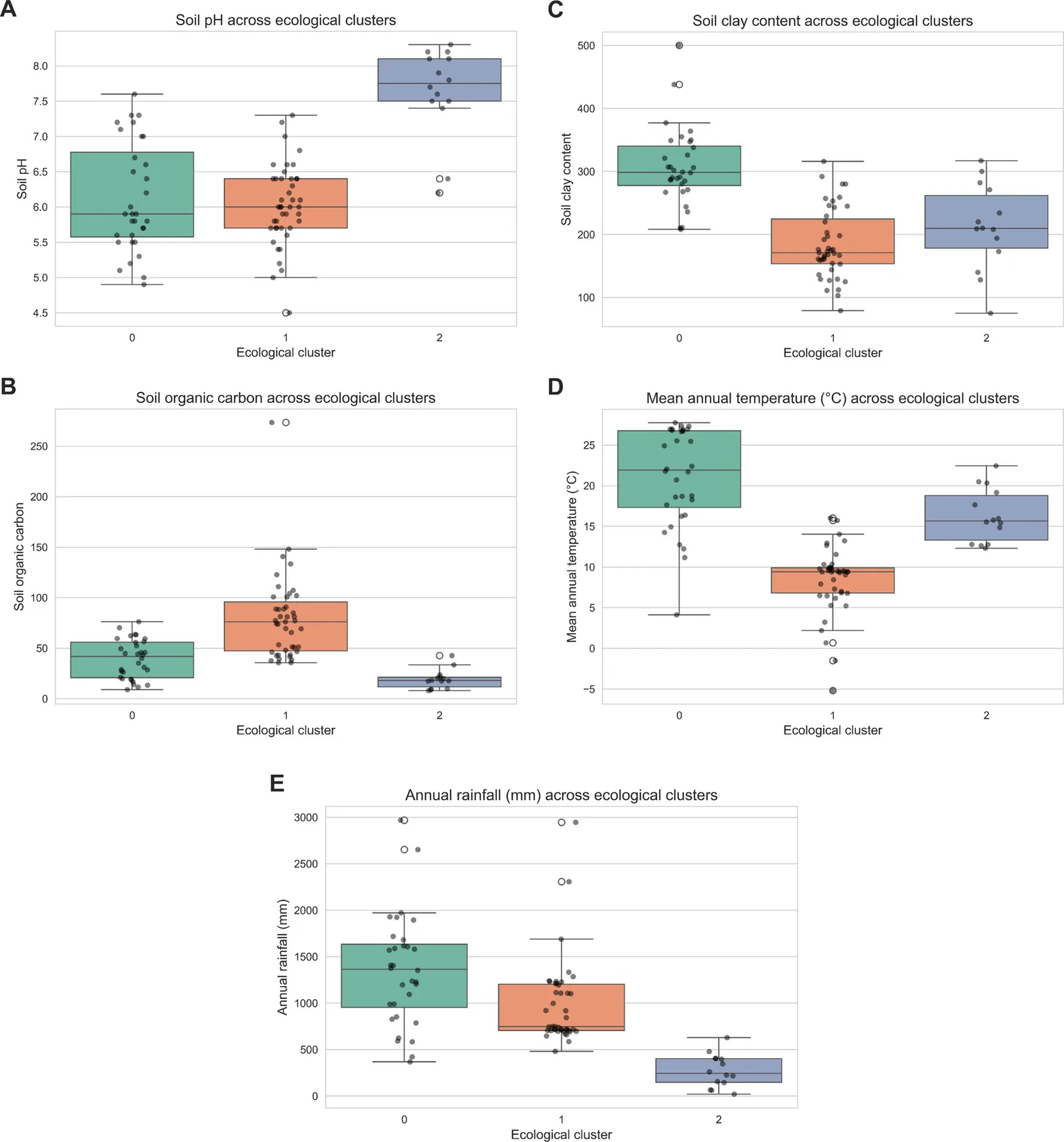
Environmental differentiation among ecological clusters identified from site-level *Streptomyces* environmental records (*n* = 89 ecological sites). **(A)** Soil pH distribution across ecological clusters. **(B)** Soil organic carbon variation among clusters. **(C)** Soil clay content across ecological groups. **(D)** Mean annual temperature differences among clusters. **(E)** Annual rainfall variation across ecological clusters. Differences among ecological clusters were evaluated using Kruskal–Wallis tests, and *p*-values were adjusted for multiple testing using the Benjamini–Hochberg false discovery rate (FDR) procedure.

Statistical analyses confirmed that all retained environmental variables differed significantly among ecological clusters (Table 3). The strongest differentiation was observed for temperature, soil organic carbon, and soil clay content, whereas rainfall and soil pH also exhibited substantial inter-cluster variation.

**Table 3:** Kruskal–Wallis analysis of environmental differentiation among ecological clusters.

Variable	Kruskal statistic	Adjusted *p*-value
Soil pH	28.25	7.36 × 10^−7^
Soil organic carbon	44.58	4.41 × 10^−10^
Soil clay	44.10	4.41 × 10^−10^
Temperature	56.61	2.55 × 10^−12^
Rainfall	40.15	2.39 × 10^−9^

### 3.4 Biosynthetic gene cluster profiles across *Streptomyces* genomes

Genome mining analysis identified extensive biosynthetic diversity across environmentally associated *Streptomyces* genomes. Using the GECCO prediction framework, a total of 363 genomes were successfully processed for biosynthetic gene cluster (BGC) detection and classification. Predicted clusters encompassed multiple major biosynthetic categories, including polyketides, non-ribosomal peptides (NRPs), RiPPs, terpenes, saccharides, alkaloids, and several hybrid biosynthetic architectures combining features of multiple metabolite classes.

Substantial variation in total biosynthetic richness was observed among genomes. Several assemblies contained fewer than 30 predicted BGCs, whereas others exceeded 70 biosynthetic regions, indicating marked differences in encoded secondary metabolic capacity across environmentally associated strains. Polyketide-associated clusters represented the most abundant biosynthetic category throughout the dataset, followed by NRPs and RiPP-associated regions. Terpene clusters were also consistently detected, although their abundance remained comparatively moderate across most genomes.

To evaluate the reliability of the predicted biosynthetic gene clusters, the confidence metrics generated by GECCO were examined across the complete dataset. The mean prediction probability (average_p) was 0.946, whereas the mean maximum prediction probability (max_p) reached 0.980. Overall, 85.6% of predicted clusters exhibited an average prediction probability of at least 0.90, 59.2% exceeded 0.95, and 70.4% reached a maximum prediction probability of at least 0.99 (Table 4). These results indicate that the majority of predicted biosynthetic regions were supported by high-confidence predictions, providing a robust basis for the subsequent ecological-genomic analyses.

**Table 4:** Summary of genome mining and biosynthetic prediction results.

Feature	Value
Total genomes screened	364
Genomes successfully analyzed by GECCO	363
Total predicted BGCs	15,839
Environmentally resolved genomes	208
Ecological clusters identified	3
Major BGC classes detected	Polyketide, NRP, RiPP, Terpene, Saccharide, Alkaloid
Hybrid biosynthetic classes detected	Yes
Maximum observed BGC richness	71
Genome size range	~5.8–13.6 Mb
Mean prediction probability (average_p ± SD)	0.946 ± 0.043
Mean maximum prediction probability (max_p ± SD)	0.980 ± 0.040
Predicted BGCs with average_p ≥ 0.90	85.6%
Predicted BGCs with average_p ≥ 0.95	59.2%
Predicted BGCs with max_p ≥ 0.99	70.4%

Integration of GECCO-derived biosynthetic predictions with curated ecological metadata produced a final ecological-genomic matrix comprising 208 environmentally resolved genomes distributed across three ecological clusters. This merged dataset enabled direct comparison between environmental structuring and genome-resolved biosynthetic profiles in downstream analyses. Genome size estimation further revealed substantial variation in assembly lengths across the analyzed strains, ranging from compact genomes below 6 Mbp to expanded assemblies exceeding 13 Mbp. Because secondary metabolism in *Streptomyces* is frequently associated with large accessory genomic regions.

### 3.5 Ecological differentiation of biosynthetic profiles

Integration of genome-resolved biosynthetic predictions with ecological clustering revealed significant variation in biosynthetic richness across environmentally associated *Streptomyces* groups (Figure 7). Although substantial overlap among genomes remained evident, differences in total BGC abundance were consistently observed across ecological clusters. Genomes associated with Cluster 0 generally displayed elevated biosynthetic richness relative to the remaining ecological groups, whereas Cluster 2 exhibited comparatively reduced total BGC counts across much of the observed distribution.

**Figure 7:**
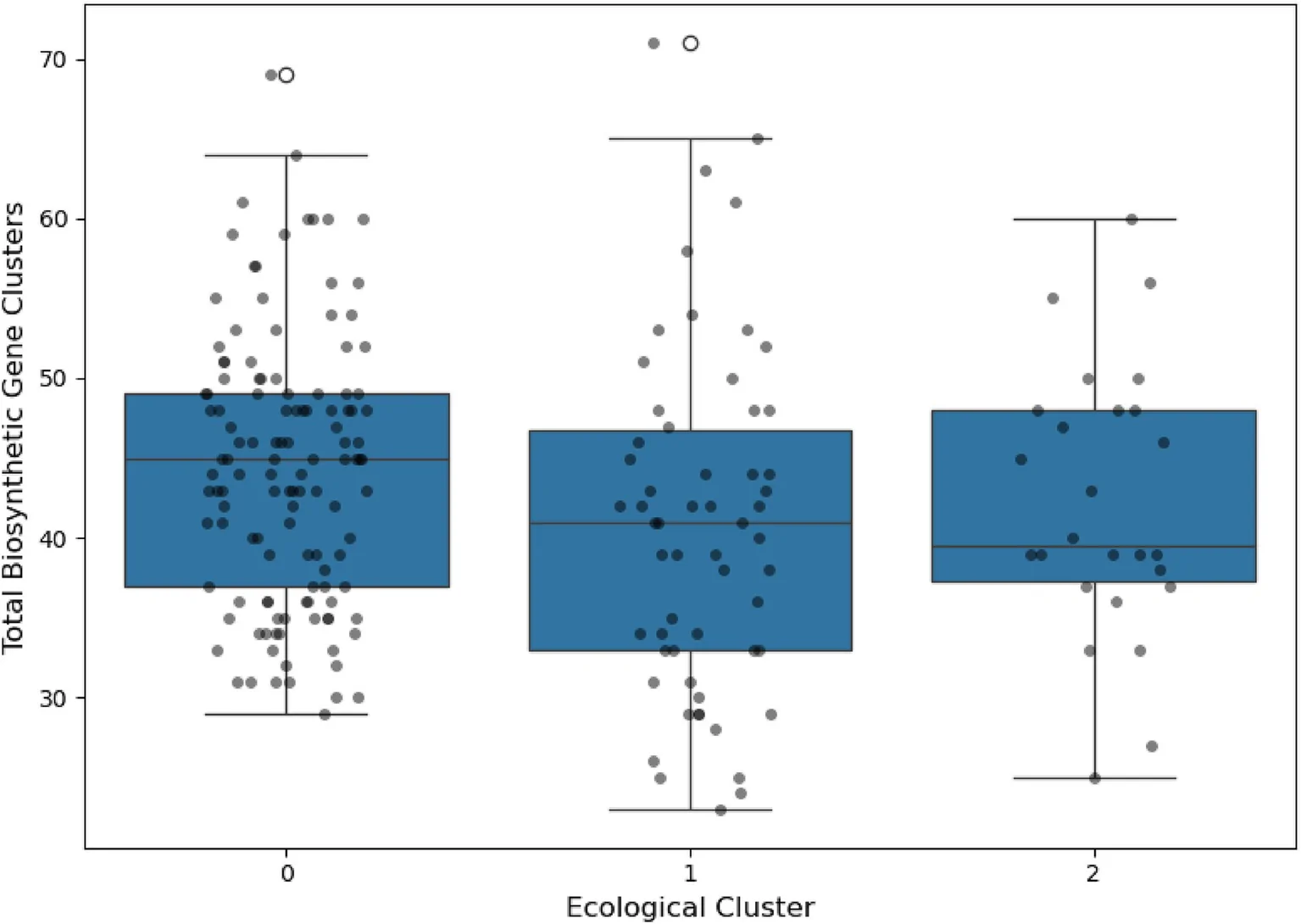
Biosynthetic gene cluster richness across ecological clusters.

Statistical analysis supported the presence of significant inter-cluster variation in biosynthetic richness (Kruskal–Wallis statistic = 7.28, p=0.026 ).

Comparison of class-specific biosynthetic profiles further demonstrated that ecological differentiation extended beyond total BGC abundance (Figure 8). Polyketide-associated clusters represented the dominant biosynthetic category across all ecological groups and consistently accounted for the largest proportion of predicted secondary metabolic regions. Non-ribosomal peptide clusters were also broadly distributed, whereas RiPP and terpene-associated regions displayed comparatively moderate abundance across the dataset.

**Figure 8:**
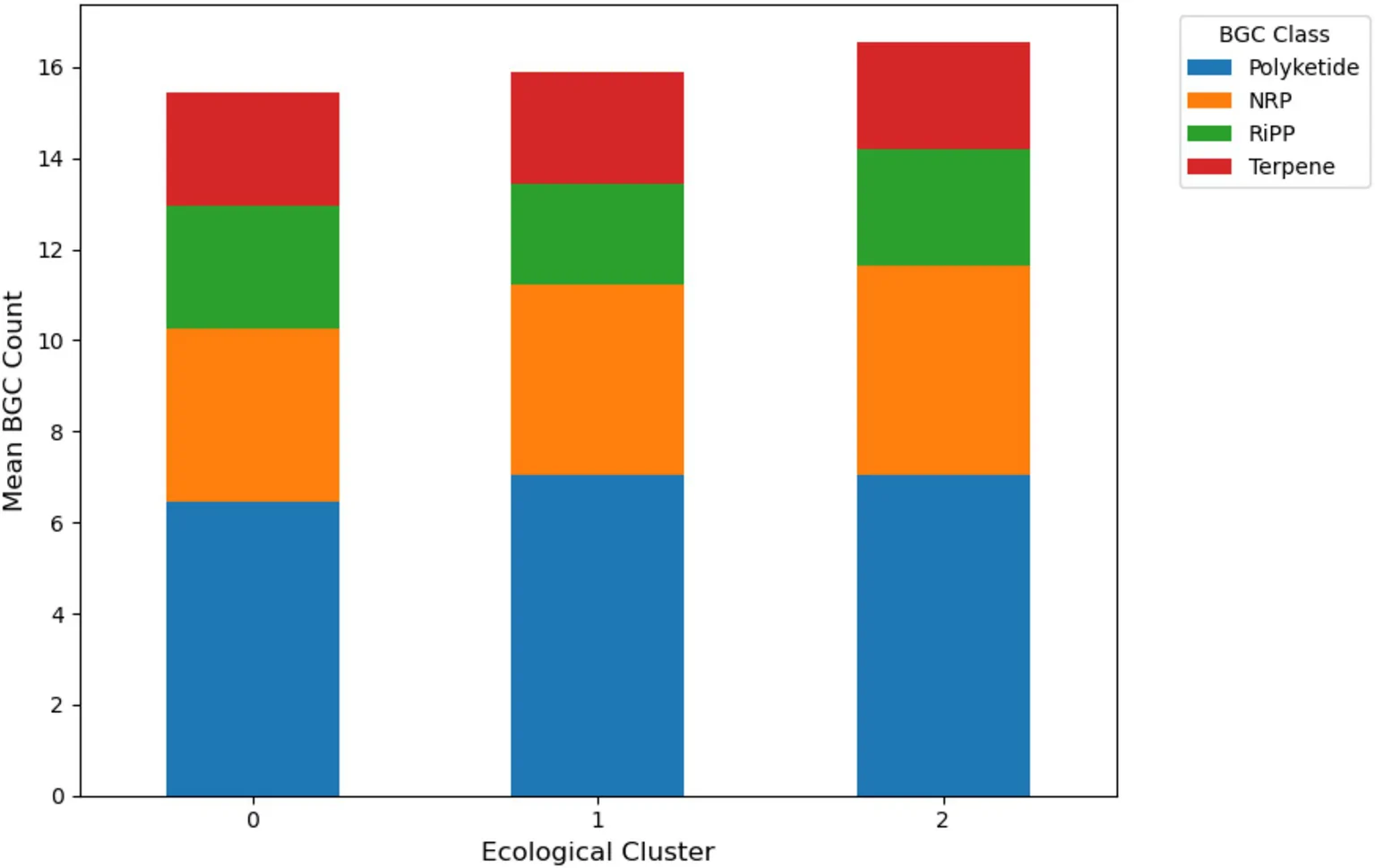
Biosynthetic class composition across ecological clusters.

Although the overall biosynthetic architecture remained partially conserved among ecological groups, subtle shifts in class composition were detectable across clusters. Cluster 2 exhibited slightly elevated NRP-associated abundance, whereas RiPP distributions varied modestly among environmental groups.

Relationships between genome size and biosynthetic composition further reinforced the ecological-genomic structuring observed across the dataset (Figure 9). Larger genomes generally contained increased numbers of predicted biosynthetic regions, particularly within polyketide-associated categories. In contrast, terpene- and RiPP-associated clusters showed comparatively limited variation across genome sizes. Genomes within Cluster 0 generally displayed larger genomes and higher BGC richness, whereas Cluster 2 was more often associated with reduced genome size and lower biosynthetic abundance.

**Figure 9:**
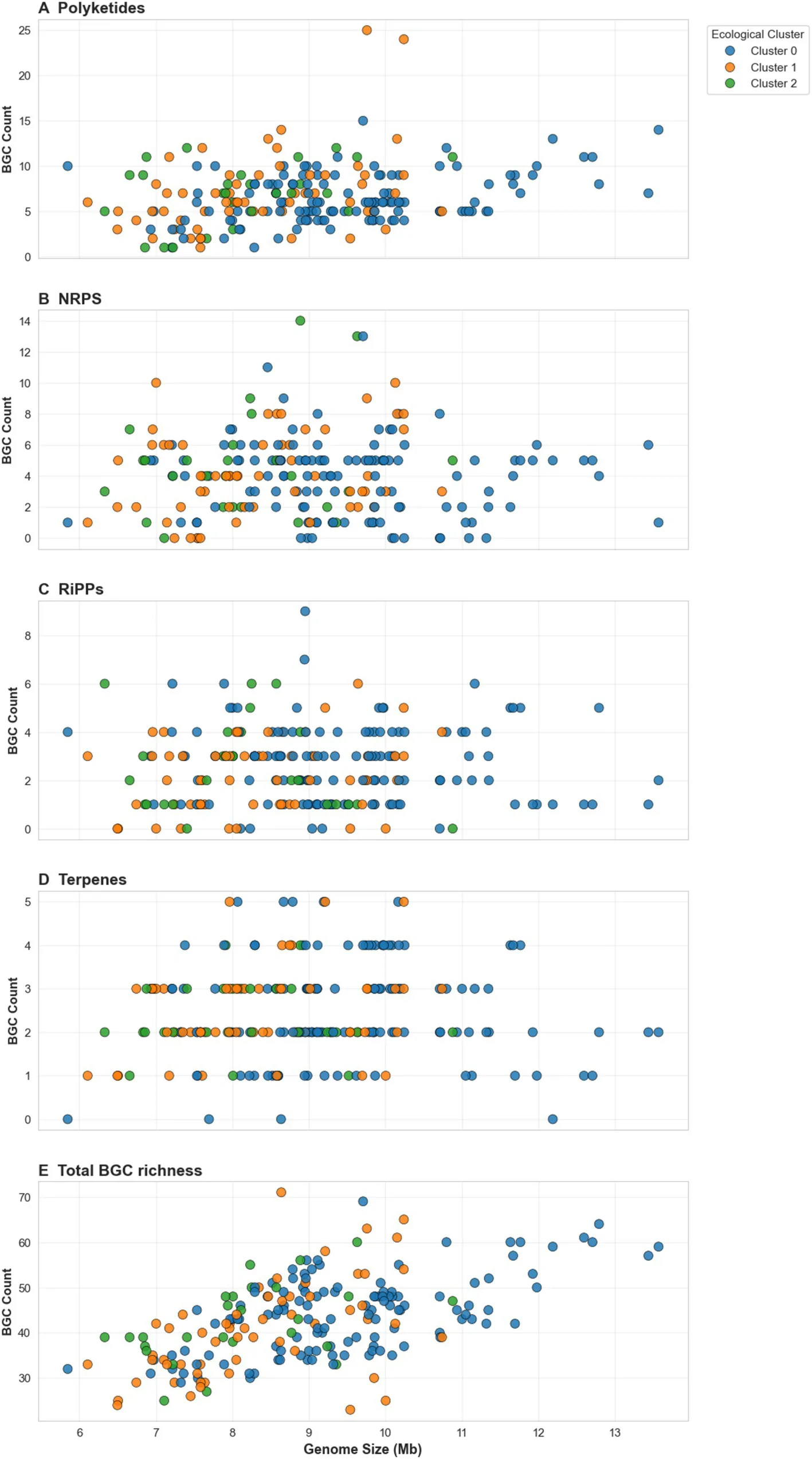
Ecological structuring of biosynthetic architectures across *Streptomyces* genomes. Panels show relationships between genome size and abundances of **(A)** polyketide-associated clusters, **(B)** NRP-associated clusters, **(C)** RiPP-associated clusters, **(D)** terpene-associated clusters, and **(E)** total biosynthetic gene cluster richness across ecological groups.

## 4 Discussion

The environmental dataset revealed substantial climatic and edaphic heterogeneity across environmentally associated *Streptomyces* habitats, particularly for rainfall, soil organic carbon, temperature, and soil pH (Table 2; Figures 1–3). PCA ordination further demonstrated that ecological sites were structured along coordinated environmental gradients rather than randomly distributed across multivariate space (Figures 2, 3). The separation observed among ecological groups following K-means clustering additionally supports the presence of distinct environmental assemblages associated with different combinations of climatic and soil-associated variables (Figures 4–6).

Genome mining analyses identified extensive biosynthetic diversity throughout the analyzed genomes. Polyketide- and non-ribosomal peptide-associated clusters consistently represented the dominant biosynthetic categories, whereas RiPP- and terpene-associated regions displayed comparatively lower abundance across most strains (Figures 7–9). The predominance of polyketide-associated biosynthetic gene clusters is particularly noteworthy given the exceptional structural diversity and broad ecological functions of polyketide metabolites. Beyond their importance as a major source of bioactive natural products, these compounds contribute to microbial competition, environmental adaptation, and interspecies communication. The patterns observed in the present study suggest that biosynthetic potential is not uniformly distributed across *Streptomyces* populations, but instead varies across environmentally distinct habitats. Although individual polyketide synthase (PKS) classes were not examined in detail, the observed ecological partitioning indicates that different habitats may represent unique sources of polyketide-associated biosynthetic diversity, providing promising targets for future natural product discovery and functional characterization. Consistent with this observation, considerable variation in total BGC richness was detected among genomes, with some strains encoding substantially expanded biosynthetic repertoires relative to others.

Assessment of the GECCO confidence metrics indicated that the predicted biosynthetic gene clusters were generally supported by high prediction probabilities across the complete dataset. The majority of predicted biosynthetic gene clusters exhibited high confidence scores, with a mean prediction probability of 0.946 and 85.6% of predicted clusters showing an average prediction probability ≥ 0.90. Furthermore, the mean maximum prediction probability reached 0.980, with 70.4% of clusters attaining maximum confidence values ≥ 0.99. Although computational predictions cannot replace experimental validation, the high confidence scores obtained across the complete dataset support the robustness of the reported biosynthetic patterns. Future studies integrating complementary genome-mining approaches, including antiSMASH, together with experimental validation will further refine biosynthetic gene cluster characterization.

The ecological and genomic analyses indicate that environmentally associated *Streptomyces* populations are structured not only across climatic and edaphic gradients but also with respect to their encoded biosynthetic architectures. Although the observed ecological separation does not imply direct environmental determination of specialized metabolism, the consistent association between ecological clustering and biosynthetic richness suggests that environmental differentiation contributes to shaping genome-scale metabolic organization across natural populations (Schniete and Fernández-Martínez, 2024). These findings support the hypothesis that distinct ecological niches harbor *Streptomyces* populations with different biosynthetic capacities, highlighting the value of integrating environmental metadata with genome mining for identifying ecological reservoirs of specialized metabolic diversity.

Beyond influencing biosynthetic gene cluster composition, environmental differentiation may also affect the activation and expression of cryptic biosynthetic gene clusters that remain silent under standard laboratory conditions. Environmental factors such as nutrient availability, microbial competition, temperature, pH, and other ecological stresses have been reported to act as regulatory signals that influence secondary metabolism in *Streptomyces*. Consequently, the ecological differentiation observed in the present study may not only contribute to variation in biosynthetic gene cluster content but may also influence the environmental conditions under which these pathways are activated and expressed (Barka et al., 2015; Rutledge and Challis, 2015; Murtaza et al., 2025).

Our findings are consistent with previous studies demonstrating substantial variation in biosynthetic gene cluster repertoires among *Streptomyces* genomes and highlighting the important roles of genome evolution, ecological adaptation, and lineage diversification in shaping specialized metabolism (Deng et al., 2025; Hariharan et al., 2025; Lorková et al., 2025; McDonald and Currie, 2017). However, whereas previous investigations primarily focused on comparative genomics or phylogenetic relationships, the present study integrates large-scale environmental metadata with genome-wide biosynthetic predictions to examine how ecological differentiation is associated with biosynthetic organization across environmentally associated *Streptomyces* populations. This ecological-genomic framework provides a complementary perspective for identifying environmentally distinct microbial populations with enhanced potential for specialized metabolite discovery and future biotechnological applications.

## 5 Conclusion

This study presents a large-scale ecological-genomic framework for investigating the relationship between environmental differentiation and biosynthetic organization across terrestrial soil-associated *Streptomyces* populations. By integrating environmental metadata, multivariate ecological analyses, and genome-wide BGC predictions, we identified substantial ecological heterogeneity and variation in biosynthetic richness and architecture across distinct environmental groups. Importantly, this approach extends conventional genome mining by incorporating ecological context into the analysis of biosynthetic diversity and may help prioritize environmentally distinct microbial populations for future functional characterization and natural product discovery. From a natural product discovery perspective, this ecology-guided framework may also support the prioritization of *Streptomyces* populations with enhanced biosynthetic potential for subsequent functional screening. Such prioritization could contribute to future efforts to identify novel bioactive compounds, including antimicrobial metabolites, which is particularly relevant given the continuing need for new antibiotics and the growing challenge of antimicrobial resistance. Given the ecological importance of *Streptomyces* in soil environments, this framework may also contribute to the identification of microbial resources relevant to biological control, soil health, and other ecosystem services supporting sustainable agriculture. Overall, these findings provide a foundation for future studies integrating ecological information, comparative genomics, and experimental validation to better characterize microbial biosynthetic diversity and its potential applications.

## Data Availability

The original contributions presented in the study are included in the article/Supplementary material, further inquiries can be directed to the corresponding author.

## Supplementary material

SUPPLEMENTARY FIGURE S1 Distribution of annual rainfall across ecological records included in the analysis.

SUPPLEMENTARY FIGURE S2 Distribution of soil pH across ecological records included in the analysis.

SUPPLEMENTARY FIGURE S3 Distribution of soil organic carbon across ecological records included in the analysis.

SUPPLEMENTARY FIGURE S4 Distribution of mean annual temperature across ecological records included in the analysis.
